# Non-parametric combination analysis of multiple data types enables detection of novel regulatory mechanisms in T cells of multiple sclerosis patients

**DOI:** 10.1038/s41598-019-48493-7

**Published:** 2019-08-19

**Authors:** Sunjay Jude Fernandes, Hiromasa Morikawa, Ewoud Ewing, Sabrina Ruhrmann, Rubin Narayan Joshi, Vincenzo Lagani, Nestoras Karathanasis, Mohsen Khademi, Nuria Planell, Angelika Schmidt, Ioannis Tsamardinos, Tomas Olsson, Fredrik Piehl, Ingrid Kockum, Maja Jagodic, Jesper Tegnér, David Gomez-Cabrero

**Affiliations:** 1 Unit of Computational Medicine, Department of Medicine, Solna, Center for Molecular Medicine, Karolinska Institutet, Stockholm, Sweden; 2 Science for Life Laboratory, Solna, Stockholm, Sweden; 3 Department of Clinical Neuroscience, Center for Molecular Medicine, Karolinska Institutet, Stockholm, Sweden; 4 Institute of Chemical Biology, Ilia State University, Tbilisi, Georgia; 5 Gnosis Data Analysis PC, Heraklion, Greece; 6 Computer Science Department, University of Crete, Heraklion, Crete, Greece; 7 Computational Medicine Center, Thomas Jefferson University, 1020 Locust Street, Philadelphia, PA 19107 USA; 8 Institute for Immunology, Biomedical Center, Ludwig-Maximilians-Universität, München, 82152 Planegg-Martinsried, Germany; 9 Biological and Environmental Sciences and Engineering Division, Computer, Electrical and Mathematical Sciences and Engineering Division, King Abdullah University of Science and Technology, Thuwal, Saudi Arabia; 10 Mucosal and Salivary Biology Division, King’s College London Dental Institute, London, SE1 9RT United Kingdom; 11 Translational Bioinformatics Unit, Navarrabiomed, Complejo Hospitalario de Navarra (CHN), Universidad Pública de Navarra (UPNA), IdiSNA, Pamplona, Spain

**Keywords:** Neuroimmunology, Data integration

## Abstract

Multiple Sclerosis (MS) is an autoimmune disease of the central nervous system with prominent neurodegenerative components. The triggering and progression of MS is associated with transcriptional and epigenetic alterations in several tissues, including peripheral blood. The combined influence of transcriptional and epigenetic changes associated with MS has not been assessed in the same individuals. Here we generated paired transcriptomic (RNA-seq) and DNA methylation (Illumina 450 K array) profiles of CD4+ and CD8+ T cells (CD4, CD8), using clinically accessible blood from healthy donors and MS patients in the initial relapsing-remitting and subsequent secondary-progressive stage. By integrating the output of a differential expression test with a permutation-based non-parametric combination methodology, we identified 149 differentially expressed (DE) genes in both CD4 and CD8 cells collected from MS patients. Moreover, by leveraging the methylation-dependent regulation of gene expression, we identified the gene *SH3YL1*, which displayed significant correlated expression and methylation changes in MS patients. Importantly, silencing of *SH3YL1* in primary human CD4 cells demonstrated its influence on T cell activation. Collectively, our strategy based on paired sampling of several cell-types provides a novel approach to increase sensitivity for identifying shared mechanisms altered in CD4 and CD8 cells of relevance in MS in small sized clinical materials.

## Introduction

Multiple Sclerosis (MS) is a complex autoimmune disease characterized by demyelination and neurodegeneration^1,2^. In most cases MS initially presents as a relapsing-remitting (RR) disease characterized by episodes of clinical symptoms followed by partial or complete recovery. With time a majority of MS patients will convert to a secondary progressive (SP) disease course, with a continuous decline in neurological functions. Although the precise triggering mechanisms remain unknown, the causal role of the immune system in MS is supported by genome-wide association studies (GWAS), which have uncovered many MS risk alleles in immune related loci^3–6^. Several disease modulatory treatments (DMTs) are now available for RR, while treatment options in progressive MS remains very limited^2^.

CD4+ and CD8+ T-cells (CD4, CD8) play a prominent role in triggering and sustaining the dysregulated immune reaction that drives disease processes in RR. Myelin-specific T cells were shown to be in a higher proportion in MS patients vs healthy controls^7,8^. HLA class II genes that are essential for the initiation of the antigen-specific immune response by CD4 cells are established to be the most important risk genes contributing to susceptibility for MS. They are essential for antigen presentation to the T cell receptor (TCR). The strongest known genetic risk factor for MS is HLA-DRB1*15:01^9^. In addition, several studies show qualitative differences in CD4 cells between controls and MS with regard to cytokine profiles and activation of myelin specific CD4 cells^10^. Regarding the role of CD8 cells, HLA class I genes required for the antigen-specific immune response by CD8 cells are shown to have protective variant (HLA-A*02:01) in MS^11^. CD8 cell infiltrates are prominent in brain MS lesions. Results from TCR sequencing of brain infiltrating CD8 cells showed a small number of clones accounting for up to 35% of CD8 suggesting clonal expansion in MS^12^.

Transcriptomic profiling allows for unbiased detection of gene expression and has been used in complex diseases such as MS to define dysregulated pathways, pinpoint candidate molecules for therapeutic interventions and cluster patients into distinct groups^13,14^. In addition, genome wide methylation arrays allow for detection of epigenetic dysregulation which affects chromatin unwinding and transcription factor binding, which in turn regulates gene expression. However, when performing molecular analysis of clinical samples, we are faced with several challenges^15,16^ such as limited sample number and a desire to do deep molecular profiling on several levels which often affects the power of detection of regulatory changes. The integration of data of different types across different samples provides a promising approach to circumvent some of these hurdles^17–19^. Here, using sorted CD4 and CD8 cells from RR and SP patients and healthy controls (HC), we perform transcriptomic profiling (RNA-Seq). Furthermore, we integrate this data at the epigenetic level by incorporating methylation profiles from the same cell-types in the same individuals. However, due to limited number of samples and limited statistical power, we adapted a *non-parametric combination analysis* framework^20,21^ to integrate such data across cell-types, and across different data-types.

## Results

### Transcriptional profiles of CD4 and CD8 cells unveil shared active genes

We analyzed samples that included CD4 from 12 HC, 12 RR and 10 SP MS patients and CD8 from 15 HC, 11 RR and 8 SP (Table 1, Supplementary Table 1).

**Table 1:** Characteristics of healthy controls and multiple sclerosis (MS) patients used for transcriptomic and paired methylation analysis in CD4+ and CD8+ T cells.

Characteristics	HC		RRMS		SPMS	
	CD4	CD8	CD4	CD8	CD4	CD8
Age (yr) mean	40.1	35.3	35.6	36.3	52.4	52.0
	(R: 27–62)	(R: 20–62)	(R: 26–46)	(R: 26–46)	(R: 35–63)	(R: 35–63)
Gender (F/M)	7/5	9/6	6/5	5/5	8/3	6/3
EDSS median			1.7	1.5	6.2	6.0
			(R: 0.5–5.0)	(R: 0.5–5.0)	(R: 5.0–8.0)	(R: 5.0–8.0)
MSSS mean			3.05	2.46	6.17	6.26
			(R: 0.67–5.87)	(R: 0.67–5.87)	(R: 5.43–8.75)	(R: 2.82–8.75)
No. of Samples	12	15	12	11	10	8
**(RNA Seq)**						
No. Of Common Samples	9	9	9	9	7	3
**(RNA Seq and Methylation)**						

HC: Healthy Control, RRMS: Relapse Remitting Multiple Sclerosis; SPMS: Secondary Progressive Multiple Sclerosis; yr: Year;

R: Range; F: Female; M: Male; EDSS: Expanded Disability Status Scale; MSSS: Multiple Sclerosis Severity Score.

Upon transcriptomic profiling (RNA-Seq) and principal component analysis (PCA) of all samples using the filtered genes (see Methods), the samples segregated according to the CD4 and CD8 transcripts. However, there was no clustering of patients based on disease states (Supplementary Fig. 1).

**Figure 1:**
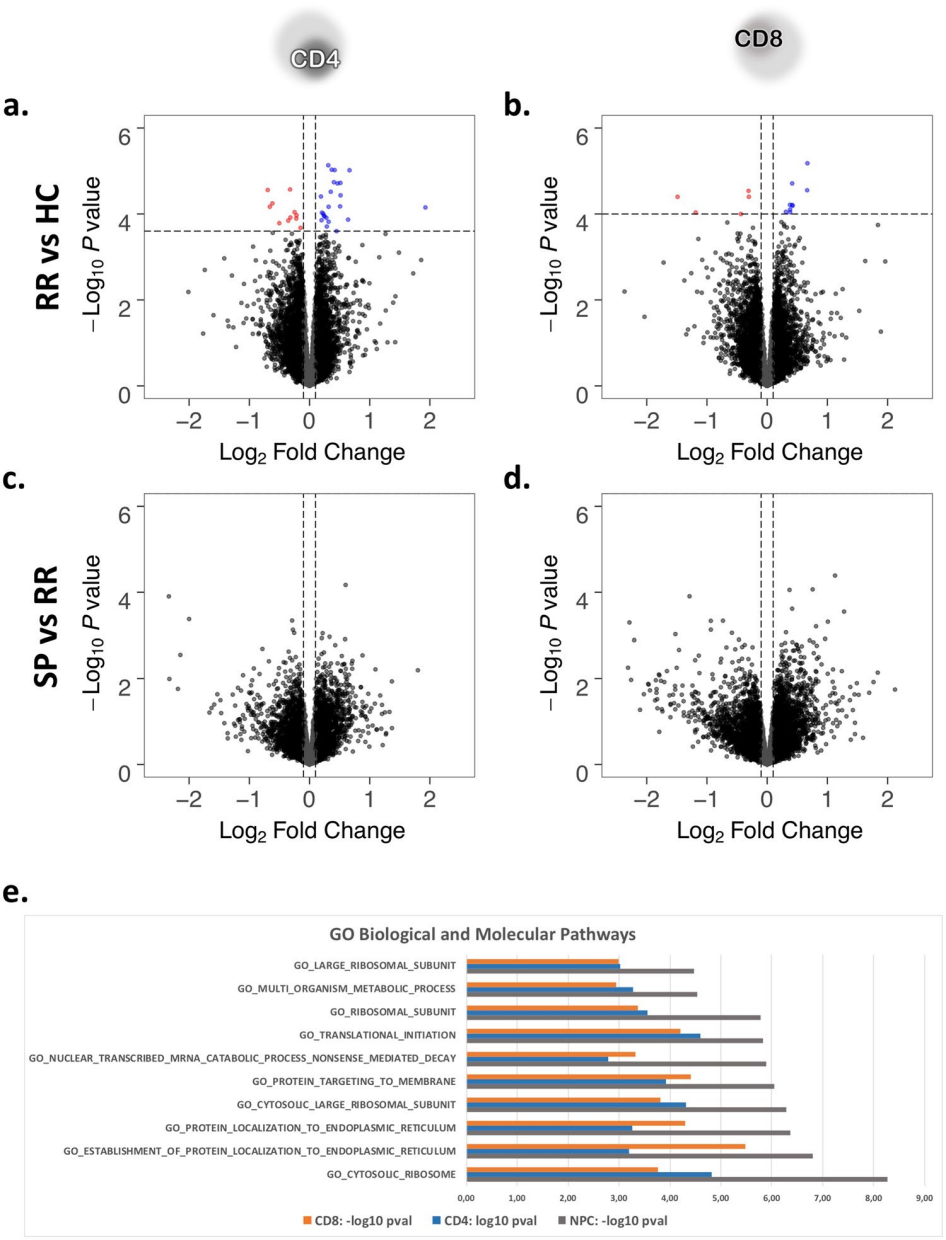
Volcano plots showing differentially expressed (DE) genes between HC and RR in CD4 (**a**) and CD8 (**b**), and between RR and SP in CD4 (**c**) and CD8 (**d**). DE analysis was performed using linear models that, in addition to disease status included age and gender as covariates (Methods). Genes passing an FDR threshold of 0.1 have been highlighted with blue if upregulated in RR (**a**) or SP (**b**) and red if downregulated in RR (**a**) or SP (**b**). (**e**) Top 10 enriched gene sets in HC-RR contrast identified by rank based gene-set enrichment when using the statistics derived from the differential expression analysis and NPC; note that the top gene-sets identified were the same in both CD4, CD8 and NPC.

Performing a differential expression (DE) analysis (FDR < 0.1) in CD4, we identified 34 genes differentially expressed between HC and RR (mean logFC of ±0.4) (Fig. 1a). In CD8, we identified 14 genes between HC and RR (mean logFC of ±0.55) (Fig. 1b). No genes could be detected in the transition from RR to SP in CD4 or CD8 using the same threshold (Fig. 1c,d).

Rank based gene-set enrichment of HC-RR in CD4 and CD8 showed enrichment for gene-sets associated with translation. Importantly, the top ten enriched gene-sets in CD4 and CD8 were the same (Fig. 1e). RR-SP showed no enrichment in CD4 and CD8.

### Disease stage-specific molecular profiles derived from non-parametric integrative analysis of cell-specific transcriptional profiles

Motivated by the finding of shared regulatory activity, such as top 10 shared enriched gene-sets in CD4 and CD8 in HC-RR, we reasoned that integrating differential analysis from both cell types could mitigate the effect of low sample number. Biologically, the integration is supported by the argument that CD4 and CD8 cells retain some common gene regulatory programs due to their recent common lineage originating from double positive thymocytes to single positive CD4^+^/CD8^−^ and CD4^−^/CD8^+^ T cells^22^. Moreover, both CD4 and CD8 are similarly influenced by genetics and external triggers such as vitamin D and viral infections, known to play a role in MS^23^. Finally, to further support our working hypothesis of shared changes in both cell-types we estimated the shared variability between CD4 and CD8. We found two shared components between CD4 and CD8 (Supplementary Fig. 2a).

**Figure 2:**
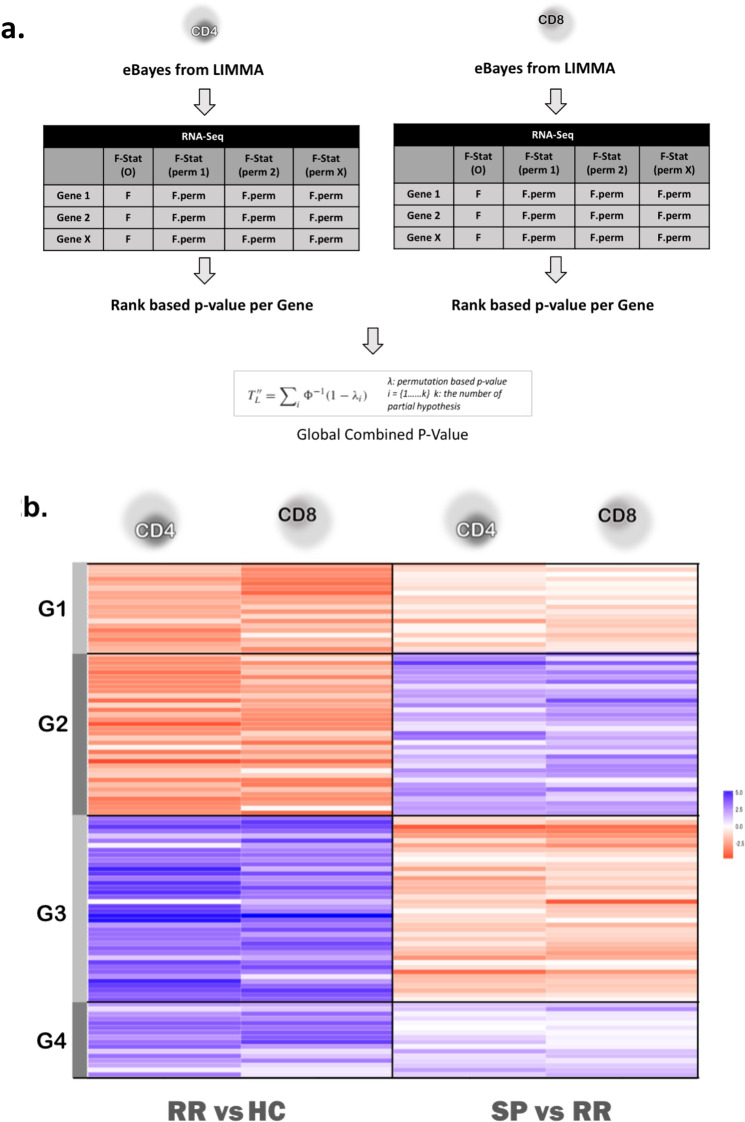
(**a**) Overview of NPC as applied to this data (see Methods). (**b**) 149 genes chosen from NPC were assigned into 5 groups to determine their shared pattern of expression. Of the 149 genes, 110 showed shared patterns of expression in RR and SP as expected from NPC. To represent directional change, here we use the t-statistic as obtained from differential expression analysis in LIMMA since the output from NPC is a global p-value lacking direction. Note: Only the 4 groups with shared patterns are represented here.

Since the sample size of our transcriptional data does not allow us to make strong statistical assumptions about the distributions we adapted a non-parametric combination (NPC) procedure for data-integration (Fig. 2a)^24^. The adaptation allowed us to use all the available samples since for some individuals either CD4 or CD8 samples were not available. To verify that our adaptation of NPC for non-perfect paired sample design was beneficial towards detecting additional shared genes, we compared the results between only-paired vs all sample analysis. Results confirmed that including all samples provided additional power and we did not identify any specific bias towards the unpaired samples (Supplementary Fig. 3).

The rank-based gene-set analysis using NPC statistics yielded the top 10 ranked pathways (q-val < 0.1) which were associated with gene translation (Fig. 1e). Reassuringly, the top pathways were the same as the pathways identified in the separate DE analysis for CD4 and CD8 (Fig. 1e). Next, we examined the 149 differentially expressed genes found to be shared by CD4 and CD8 as revealed by NPC (p-value ≤ 0.001, FDR ≤ 0.1; Fig. 2b). Importantly, while only p-values were used in the identification of DE genes, in most cases the direction of change was similar in both CD4 and CD8 analysis, supporting the hypothesis of shared mechanisms among cell types which NPC is based on.

These 149 genes could be categorized into five sub-groups (G1-G5) described as, G1: genes that were upregulated in RR (HC-RR) and SP (RR-SP), G2: up-regulated in RR (HC-RR) and down-regulated in SP (RR-SP), G3: down-regulated in RR (HC-RR) and up-regulated in SP (RR-SP), G4: down-regulated in RR (HC-RR) and SP (RR-SP) (Fig. 2b, Supplementary Table 2). G5: genes that did not follow a common pattern for HC-RR and RR-SP (Supplementary Table 2). G1 and G4 were interpreted biologically as progressive changes in expression from HC to RR to SP and primarily contained genes involved in mRNA processing and translation. G2 and G3 were interpreted as expression changing in RR, after which their expression level returned to a HC like level in SP. The associated genes included for example several genes with known immune related functions such as *BCL10*, *HLA-G*, *IGJ*, *SOX4*, *IFIT2*, *IRGM*, *F2RL1*, *STAMBP*, *GIMAP4*, small RNA regulation like *TNRC6B*, *TDRKH*, and neuronal function related *GMFB*, *NEFL*. Several of these genes have been reported earlier in the context of MS, thus confirming the biological relevance of the genes discovered here by using an integration of cell-specific transcriptional profiles.

### Correlated regulation of stage-specific differentially expressed genes via DNA methylation

**Figure 3:**
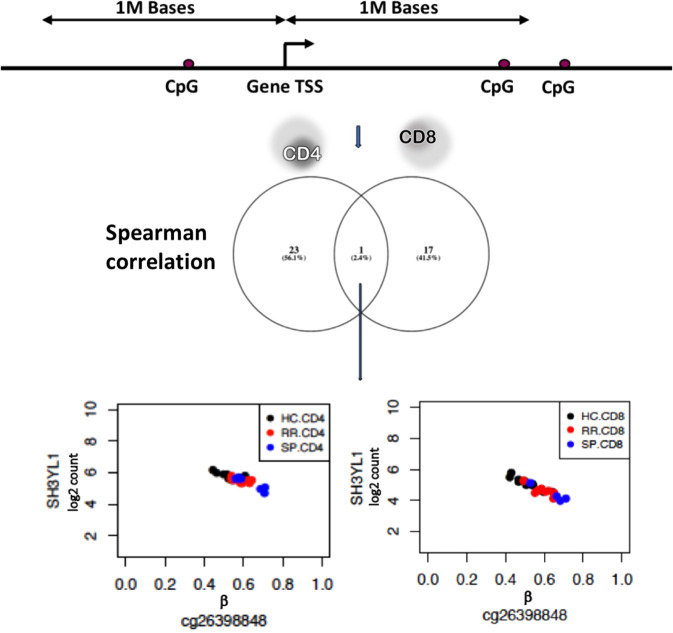
Overview of methodology used to find associations between expression and methylation results from NPC. Followed by, top ranked gene-probe pairs with a spearman correlation of <−0.5 and >0.5 and p-value < 0.05. Finally, a single pair was found in both CD4 and CD8. This pair, SH3YL1-cg26398848, showed a decrease in expression with increasing methylation from HC to RR and from RR to SP.

Differences in gene expression can be regulated by epigenetic mechanisms, such as DNA methylation. Therefore, we combined expression data with methylation profiles obtained from the same set of patients as in the present study. Similar to RNA-seq, we estimated the shared variability between CD4 and CD8 in DNA Methylation and identified one shared component. Again suggesting CD4 and CD8 share changes in DNA methylation (Supplementary Fig. 2b,c). NPC was performed on CD4 and CD8 methylation data and obtained 1838 differentially methylated probes (p-value ≤ 0.001 and FDR ≤ 0.2). Next, we overlapped these 1838 probes with 149 genes and found 360 gene-probe pairs within a distance of 1 Mb (see Methods). Of these, 24 and 18 pairs correlated in CD4 and CD8, respectively (p-value ≤ 0.05) (Fig. 3a and Supplementary Table 3). One pair was uniquely shared between CD4 and CD8. This pair, *SH3YL1*-cg26398848, had its probe in the promoter region of *SH3YL1* and ranked highest in CD4 and CD8 (Spearman correlation: −0.76 and −0.92; p-value < 4.08 × 10^−5^ and <1.61 × 10^−6^, respectively). This pair showed decreasing expression of *SH3YL1*, concordant with an increase of methylation as progressing from HC-RR-SP. Using the Ensemble regulatory build^25^, which contains cell type specific regions involved in gene regulation, we identified this probe to be in an active promoter of the *SH3YL1* gene in CD4 and CD8 (ENSR00000111917). Strong anti-correlation of promoter methylation with gene expression has been well documented as a mechanism to inhibit gene transcription^26^. In summary, our multi-layer analysis suggested that SH3YL1 plays a role in disease.

### SH3YL1 as a target of functional studies in CD4 cells and its potential role in Multiple Sclerosis

**Figure 4:**
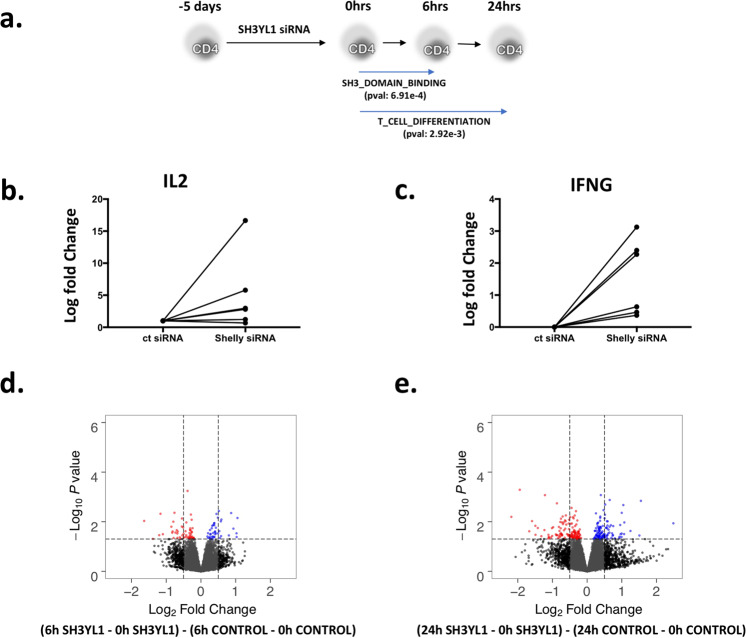
siRNA-mediated silencing of SH3YL1 in CD4+ T cells from healthy donors. (**a**) Shows the experimental overview and significantly enriched pathways. (**b**,**c**) qRT-PCR analysis of selected genes in TCR-stimulated control or SH3YL1 knockdown cells. *IL2* and *IFNG* show an increase post stimulation between Control siRNA-treated and SH3YL1 siRNA-treated cells. (**d**,**e**) Differential Expression across time (interaction) to determine the genes primarily affected by SH3YL1 silencing during activation in 2 contrasts, namely 0 hrs–6 hrs (**d**) and 0 hrs–24 hrs (**e**) with blue highlighting genes upregulated and red highlighting genes downregulated upon SH3YL1 silencing.

To assess the putative functions of *SH3YL1*, we performed siRNA-mediated knock-down experiments of *SH3YL1* in CD4 cells (Fig. 4a). There is a strong body of evidence that CD4 cells are important in MS pathogenesis based on data derived from genetics to functional animal studies^27^.

Following silencing of *SH3YL1* for 5 days, we stimulated the cells for 6 hours with TCR and costimulation. Strikingly, 6 out of 6 donors tested displayed an increase of *IFNG* expression and 4 of 6 donors in addition displayed increased *IL2* expression upon *SH3YL1* silencing compared to control siRNA (Fig. 4b,c). Whole transcriptomic profiling was done in 4 donors at three time-points; 5 days after silencing (0 hours), followed by 6 and 24 hours of stimulation (Methods). More than 70% silencing in the expression of *SH3YL1* was observed upon 5 days of silencing compared to control using qPCR. In addition, *SH3YL1* showed a decrease in expression after activation in both control and siRNA silenced groups. To determine activation induced changes upon silencing with time we analyzed two contrasts “0 hrs–6 hrs” and “0 hrs–24 hrs”. We detected 96 and 244 DE genes, respectively (p-value < 0.05) (Fig. 4d,e). Rank-based gene set enrichment showed “SH3 Domain Binding” (p-value < 0.0006) and “T cell differentiation” (p-value < 0.002) as the top-ranking gene-sets after 6 and 24 hours, respectively (Fig. 4a). This data suggests that downregulation of *SH3YL1* further promotes T cell activation, and unveils *SH3YL1* as a novel regulator of TCR-induced cytokine expression.

## Discussion

Here we report a detailed gene expression profiling analysis of CD4 and CD8 cells in blood samples from healthy controls, and clinically well-characterized MS patients including both RR and SP. Our analysis reveals 149 differentially expressed genes in both CD4 and CD8, where several genes and processes were of confirmatory nature, as well as discovering several novel genes and processes putatively involved in MS. Despite the inherent clinical and biological variability and limited number of samples, we were able to extract MS relevant signals due to our adaption of a permutation-based non-parametric combination (NPC) methodology. To focus our down-stream analysis we co-analyzed the RNA-seq data with DNA methylation data from the same patients. Thus, using such paired samples from the same individuals, we determined the role of epigenetic changes from DNA methylation and their association with changes in expression. This analysis identified the *SH3YL1* gene, which displayed significant correlated expression and methylation changes in MS patients. Finally, this observation was followed up by silencing *SH3YL1* in primary human CD4 cells, which demonstrated its influence on T cell activation and differentiation.

In perspective, previous studies have investigated gene expression using microarrays in RR and SP from whole blood or PBMCs reporting marked changes between HC, RR and SP^13,14^. However, a detailed analysis of the contribution of the different subtypes from PBMCs have not yet been performed to our knowledge. As a logical next step, we profiled CD4 and CD8 in RR and SP given their central role in the establishment of MS and subsequent progression. Clearly, sample heterogeneity associated with a complex multifactorial disease such as MS can lead to gene expression variability leading to low power of detection of DE. Excluding genetics, epigenetics and environment, some putative factors contributing to heterogeneity from the sampling include, (i) the time from relapse and the subsequent sampling which may vary due to patient availability/scheduling, which can lead to variations in cells profiled, (ii) though we considered patients who were either newly diagnosed with no prior treatment or within a 6-month wash-out period, different treatments prior to washout may have longer lasting effects, (iii) T cells are made up of multiple subsets which depending on their relative composition and relevant disease activity at the time of sampling can contribute differently to the overall expression of genes detected.

With these limitations in mind we carried out the analysis for detecting changes in different cell types and disease stages in two steps, (i) individual changes in CD4 and CD8 cells per disease stage, (ii) pooled CD4 and CD8 changes in RR and SP using NPC to increase sample number and power. Application of NPC allowed us to identify combined dysregulated molecular events in a statistically significant way. This is especially useful in cases where sample size is limiting as in the case of MS and where profiling can be expensive for larger sample numbers. In this sense, we believe that our methodology could be useful in other clinical settings investigating diseases under the constraints of sampling.

Using NPC, we identified both novel and previously reported genes in the context of MS but not all from T cells. For example, *HLA-G* or human leucocyte antigen G reported as an anti-inflammatory molecule controlling and inhibiting cell activation. Soluble HLA-G has been shown to be upregulated in cerebrospinal fluid (CSF) of RR patients. CD4 and CD8 regulatory T cells of thymic origin, express *HLA-G* potentially counteracting inflammatory autoimmune processes^28,29^. In our data we do observe *HLA-G* being upregulated in CD4 and CD8 during RR but downregulated in SP (Supplementary Fig. 4a,b, Table 2). *IGHG1* (Supplementary Fig. 4c,d) and *IGHA1* (Supplementary Fig. 4e,f) were seen to increase their expression in both CD4 and CD8 in RR but decreased in SP (Supplementary Table 2). This is striking because these genes code for immunoglobulin heavy alpha and gamma constant regions of IgA and IgG produced by B cells. We find the possibility of high B cell contamination in our samples to be unlikely since these cells were FACS sorted on CD3/CD4 and CD3/CD8 with greater than 99% purity. *IGHG1* has also been shown to be differentially expressed on the protein level in CD4 and CD8^30^. Another gene, *NEFL* codes for the neurofilament light (NfL) chain which is being studied as a potential blood and CSF biomarker of axonal damage in MS^31^. *NEFL* is found differentially expressed with a decrease in RR and increase in SP (Supplementary Fig. 4g,h, Table 2). Serum NfL has been shown to be increased both in RR and SP compared to controls and to correlate with clinical and neuroradiological outcomes^32^. While serum NfL is thought to come primarily from damaged neural tissue, this data suggests expression and similar patterns in CD4 and CD8 T cells.

Performing an overlap analysis of our top DE genes and methylated probes we identified regulatory changes leading to reduced expression of *SH3YL1* from HC-RR-SP. *SH3YL1* has been included only in a small number of studies, which suggest role(s) in actin filament formation, dorsal ruffle formation and migration, while a possible role in context of T cell function remains unexplored^33–35^. Silencing *SH3YL1* in CD4 cells followed by T cell activation showed a decrease in the expression of *SH3YL1* with time similar to the pattern we see in the MS data. This suggests that the downregulation of *SH3YL1* we observed in MS patients compared to controls is a consequence of activation of T cells. Specifically, analyzing the downstream effects of activation in *SH3YL1* silenced CD4 cells, we first noticed a small increase in *IL2* and *IFNG* upon silencing and activation. As a rule, increased IL-2 production occurs upon activation of T cells and our silencing data shows a further increase in *SH3YL1* silenced cells. This increase in turn may contribute to higher T cell activation upon 24 hours of stimulation which has previously been documented^36^. While previous reports in other cell types have stated a role for *SH3YL1* in actin filament formation, they consistently saw an upregulation of *SH3YL1*. Our findings however suggests that a decrease in *SH3YL1* leads to higher activation and differentiation of T cells. Although *SH3YL1* does not map close to any of the MS associated polymorphism^5^, trans-acting effects from remote polymorphisms may affect the epigenetic state of *SH3YL1*, in turn affecting expression patterns. Clearly, however, additional studies are needed to explore a possible role for *SH3YL1* as a driver for the dysregulated immune response in MS.

The genes, the biological processes and epigenetic alterations detected in our analysis provide an insight into the function of T cells in the development and progression of MS. Translation of these results into disease relevant knowledge remains a challenge especially for a complex disease such as MS. We herein demonstrates the advantages of integrating multiple data sets using advanced statistical methods to identify relevant pathways active in CD4 and CD8 cells in small-sized clinical materials. We also believe that this strategy will turn useful for hypothesis building, functional insights into the role of T cells and validation of findings from related studies not only in MS, but also for autoimmune diseases in general.

## Material and Methods

### Sample collection and ethics statement for transcriptomic profiling of MS patients and gene silencing of SH3YL1

Blood was collected from MS patients in accordance with the McDonald criteria from the Neurology Clinic at the Karolinska University Hospital, Solna. All patients were either newly diagnosed with no prior treatment or within a 6-month wash-out period (no medication was administered) prior to the collection of samples. Patients included 12 Relapse Remitting MS (RR) and 10 Secondary Progressive MS (SP). Blood was also collected from 17 gender matched healthy controls (HC). All study participants had given their written informed consent. Ethical approval number: 2009/2107-31/2 for MS Patients and 2010/879-31-1 for Healthy Controls. Ethical Permits were obtained from the Regional Ethical Review Board in Stockholm, Sweden (Regionala etikprövningsnämnden i Stockholm).

For SH3YL1 silencing, anonymized healthy donor buffy coats were purchased from the Karolinska University Hospital (Karolinska Universitetssjukhuset, Huddinge), Sweden and research was performed according to the national Swedish ethical regulations (ethical review act, SFS no. 2003:460).

### Isolation of T cells for transcriptomic profiling

PBMCs were isolated from 50 ml of blood drawn from MS patients and healthy controls as follows, 25 ml of blood was diluted with 10 ml of PBS (Sigma). 20 ml of this diluted blood were overlaid onto 10 ml of Ficoll (GE Healthcare). The tube was centrifuged at 440 g for 20 min. The PBMC layer was collected into ice cold PBS and centrifuged at 440 g for 7 min with brake. Red blood cells were lysed by resuspending the pelleted PBMCs in 5 ml of ACK lysis buffer (Life Technologies) and leaving them on ice for 5 min. After washing away the ACK buffer the final pellet was resuspended in 1 ml of PBS and the number of PBMCs was determined in a Burker chamber following a viability staining with Trypan Blue (Sigma). A positive selection of CD14+ cells was accomplished by adding MACS microbeads (Miltenyi Biotec) conjugated with monoclonal anti human CD14 antibodies to freshly prepared PBMCs in MACS buffer (prepared according to manufacturer’s instructions). Briefly, after incubation of the cells and microbeads (15 min at 4 C), cells were washed and resuspended with MACS buffer and loaded on top of the separation column. Unlabeled cells would pass through the column and were collected for flow cytometry sorting of CD4+, CD8+ T cells and CD19+ B cells. Sorting was done using fluorochrome conjugated antibodies against human CD3 (clone UCHT1, PE-conjugated, BD Bioscience), CD4 (clone SK3, APC-conjugated, Becton Dickinson), CD8 (clone SK1, FITC-conjugated, Becton Dickinson) for CD4+ and CD8+ T cells, and CD19 (clone SJ25C1, APC-Cy7-conjugated, Becton Dickinson) for B cells, using high speed MoFlo cell sorter with >99% purity (Beckman Coulter, Inc). Only CD4 and CD8 were used for transcriptomics and all 4 cell types were used for methylation (only CD4+ and CD8+ T cell data shown here for methylation). All CD4 and CD8 samples were not paired due to limitations in quality or quantity of RNA for transcriptomic and methylation profiling.

### Nucleic acid isolation: RNA and DNA

Total RNA was extracted with Trizol (Invitrogen) using the miRNeasy Kit (Qiagen) according to manufacturer’s recommendations, and integrity was confirmed by BioAnalyzer (RNA integrity number (RIN > 9) using the Agilent RNA 6000 Nano Kit (Cat. No. 5067-1511). Extraction of genomic DNA was carried out using a MinElute Mammalian Genomic DNA Miniprep kit (Sigma-Aldrich). The amount and purity of DNA was determined using a NanoDrop ND-1000 Spectrophotometer (NanoDrop Technologies Inc).

### Transcriptomic analysis of MS and HC data

#### RNA-seq design

To identify possible confounders during quantification that arise from variability associated with batch of library preparation and batch of sequencing, samples were stratified. Stratification was conducted considering three biological variables (cell type, disease state, gender) and two technical variables (library preparation and sequencing run). Library preparation for sequencing was done in 2 batches of 40 and 31 samples each. Sequencing of libraries was done in 2 batches with an average of 8 libraries per lane.

#### Library preparation and sequencing

Sequencing libraries were prepared from 500 ng of total RNA using the Illumina TruSeq mRNA Stranded Library Preparation Kit (Cat. No. RS-122-2103) according to the manufacturers protocol. Quality and quantity of the libraries was determined using the Agilent High Sensitivity DNA Kit (Cat. No. 5067-4626) and NanoDrop ND-1000 Spectrophotometer (NanoDrop Technologies Inc). Sequencing of libraries was carried out on the Illumina HiSeq 2500 as per the manufacturer’s instructions. ~10 Mio (between 5 and 20 Mio) 75 bp paired end reads were obtained per sample.

#### Bioinformatics QC and preprocessing

Quality of the reads was assessed before and after trimming using FastQC v0.11.4^37^. Illumina adapters and low-quality nucleotides were trimmed using Cutadapt v1.9.1^38^. Fragments were aligned to human genome (Ensembl GRCh 37) using TopHat2 v2.1.1^39^. Ht-seq was used to count reads in genes using the parameters (–m union –s reverse)^40^. Genes with greater than one read per million in all samples were included in the analysis. As a result, a total of 13,119 genes (Ensembl) were selected for the analysis.

#### Preprocessing

Conditional quantile normalization^41^ was used to correct for systematic biases associated to GC content, gene length and library size. Batch effects associated to library preparation were corrected using COMBAT^42^.

#### Differential expression

For all genes the differential expression per cell type (CD4, CD8), per contrast, RRvsHC denoted as “HC-RR” and SPvsRR denoted as “RR-SP”, was determined using LIMMA^43^. Linear models used for differential expression included as explanative variables: disease group (HC, RR or SP), age and gender. For each cell type and contrast a gene was considered significant if False Discovery Rate (FDR) < 0.1^44^.

### DNA methylation analysis

DNA methylation data was generated using the Infinium Human Methylation 450 K Bead Chip arrays. Sample processing including bisulfite conversion, was done at the bioinformatics and expression analysis core facility (BEA), Karolinska Institutet (Stockholm) for monocytes and CD4 T cells, and at Johns Hopkins University School of Medicine (Baltimore) for CD8 T cells and CD19^+^ B cells. Cases and controls were randomized on 450 K arrays.

Data was first analysed using the Minfi^45^ and ChaMP^46^ packages. Type 1 and Type 2 probes found on the array were normalized using BMIQ^47^. Probes not passing a detection p-value of 0.01, probes with known SNPs and X and Y chromosomes were filtered out. Batch correction (for slide batch) was done using COMBAT^42^.

Differential methylation per cell type (CD4, CD8), per contrast, RRvsHC denoted as “HC-RR” and SPvsRR denoted as “RR-SP”, was determined using LIMMA^43^. Linear models used for differential methylation included as explanative variables: disease group (HC, RR or SP), age and gender.

### Non-parametric combination (NPC)

The Non parametric combination (NPC) method was applied using the “omicsNPC” R function implemented in the STATegra R package^21^. NPC computes global p-values as a combination of the results of several partial tests. In our analyses we used the NPC for computing global p-values for the differential expression of each gene across both CD4 and CD8 cells datasets. NPC’s operation includes the following steps:

1. For each gene, the F statistic computed by the Limma R Package is used to assess the presence of a significant change in each of the considered cell types (CD4 and CD8 cells).
2. The null-distributions of the F statistics are estimated through a permutation approach. Disease labels (HC,RR,SP) are randomly permuted at each iteration. However, since we also had a few samples that were unpaired, the following change was incorporated. If a sample was shared between CD4 and CD8, the disease label (HC or RR or SP) associated to the permutation was the same, if a sample had only CD4 or CD8, then it would get an independent label. This would result in the correlation structure between CD4 and CD8 being preserved and measurements from un-paired samples being factored into the p-values computation while employing all available samples in the analysis.
3. Permutation-based (partial) p-values are computed for each gene separately for CD4 and CD8 cells.
4. A global statistic for each gene is obtained by combining the partial p-values from CD4 and CD8 cells with the Liptak combination function. Null distributions for the global statistics are computed by repeating the p-value combination procedure for each permutation, and global p-values are calculated by comparing the global statistics against their respective nulls. The Liptak combination function reinforces the significance of results supported by several partial p-values, and penalizes results that are significant in only a fraction of the partial tests.

Results were considered significant if they had a global p-value < 0.001 and FDR < 0.1.This resulted in 149 genes.

The same method was applied on the CD4 and CD8 cells for methylation 450 K array data. Methylated probes were considered significant if they had a global p-value < 0.0001 and FDR < 0.2. This resulted in 1838 probes.

### Principal component analysis

In order to estimate the shared variability between CD4 and CD8 we used the Joint and Individual Variation Explained (JIVE) methodology^48^. First, we computed the gene-expression derived shared components between CD4 and CD8 using the 29 overlapping samples. JIVE identified a total of two shared components and 6 (CD4) and 9 (CD8) individual components (Supplementary Fig. 2a). Secondly we computed the shared DNA Methylation components between CD4 and CD8 using the overlapping 18 samples; JIVE identified one shared component and 7 (CD4) and 5 (CD8) individual components (Supplementary Fig. 1b,c). To perform the analysis and plots the R packages r.jive and STATegRa were used.

### Gene set enrichment analysis

Gene set enrichment was assessed using GAGE^49^: in each case, a p-value was used for the ranking of the genes. Three pathway gene sets were used for gene set enrichment, GO: Biological Pathways, Immunological Pathways^50^, KEGG^51^. Gene sets below 20 genes and above 200 genes were excluded from our analysis. The Ingenuity Pathway Analysis (IPA, Qiagen) was used to determine upstream regulators and biological function using right tailed Fisher’s exact test, followed by Benjamini– Hochberg correction^44^.

### Overlap analysis of paired methylation and expression data

Methylation has been shown to affect binding of proteins to gene promoters, affect chromatin structure and determine affinity of binding of regulatory proteins such as transcription factors^52^. We selected pairs of MS genes (167) and MS CpG sites (1838) identified which were less than 1 Mb apart; for the genes we used the Transcription Starting Site (TSS) as the reference since one million bases (1 Mb) has been shown to be an upper limit for detecting cis acting enhancers while also being able to determine closer cis acting regulatory elements^53^. We identified 334 gene-probe pairs. We computed spearman correlation for those pairs, using only samples that had data for both methylation and gene expression (9HC, 9RR, 7SP for CD4 and 9HC, 9RR, 3SP for CD8). Using spearman correlation, the expression and methylation of all (HC,RR,SP) for a given gene-probe pair was assessed separately in CD4 and CD8 cells. Probes were ranked based on p-value of correlation separately for CD4 and CD8.

### siRNA based gene silencing of SH3YL1: nucleofection and T cell activation

PBMCs were isolated according to standard Ficoll density gradient procedures from healthy donor buffy coats. CD4+ CD25− T cells were then isolated by negative magnetic isolation using human CD4 T cell isolation kit and CD25 microbeads (Miltenyi Biotec). 12 Mio CD4+ CD25− T cells from individual donors were resuspended in 100 μl of Nucleofection® buffer solution for human primary T cells (Nucleofector™ Kits for Human T Cells, Lonza) containing 2 µM of ON-TARGETplus SH3YL1 siRNA pool or ON-TARGETplus non-targeting control pool (Dharmacon, GE). The cells were transfected using program U-014 of the Nucleofector™ 2b device using manufacturer’s recommendations. Following nucleofection, the cells were transferred to pre-warmed X-VIVO 15 medium (Lonza) and incubated for 4.5 days. The medium was changed following 5 hours of incubation meanwhile. The cells were equally distributed for 3 time points; resting, 6 hours and 24 hours. The cells for the later time points were stimulated with antibody against CD3 (0.2 µg/ml, clone OKT3; Biolegend, LEAF grade; cat. no. 317315), antibody against CD28 (2 µg/ml, clone 15E8, Miltenyi Biotec, functional grade, cat no 130-093-375), and goat anti-mouse Ig antibody as a cross-linker (2 µg/ml, SouthernBiotech, cat no. 1010-01) mimicking TCR and co-stimulation for the afore mentioned time periods at 37 °C and 5% CO_2_.

### RNA Preparation and qRT-PCR

RNA was extracted using the AllPrep DNA/RNA/Protein Mini Kit (Qiagen), quantified using the Nanodrop 2000 (Thermo Scientific). cDNA was prepared using the SuperScript VILO cDNA Synthesis Kit (Invitrogen) according to the manufacturer’s instructions. mRNA was quantified using Taqman probes (Applied Biosystems best coverage probes for *IL2*, *IFNG*, *RPL13A* and *SH3YL1*; FAM reporter) with the Taqman gene expression master mix (Applied Biosystems). The relative mRNA expression was determined by normalization to *RPL13A*.

Note: Same RNA was used for qRT-PCR and Transcriptomics.

### Transcriptomic Analysis of siRNA Silenced Samples

Samples were prepared and analyzed similar to the MS patient transcriptomic data with the following changes. Stratification considered: donor, time point of stimulation and type of siRNA. Library preparation for sequencing was done in a single batch. Sequencing of libraries was done in 3 lanes with 9–10 libraries per lane on the Illumina HiSeq 2500 as per the manufacturer’s instructions. Between 10 and 20 Mio 100 bp paired end reads were obtained per sample. Genes with greater than one read per million in more than 3 samples were included in the analysis, leaving 12,420 genes (Ensembl) for downstream analysis. Batch correction for sequencing batch was done using COMBAT.

Differential expression was carried out using LIMMA with a design that models group specific (siRNA and control) differences (interaction term). Namely, (6 h SH3YL1 - 0 h SH3YL1) - (6 h CONTROL - 0 h CONTROL) denoted at “0 hrs–6 hrs” and (24 h SH3YL1-0 h SH3YL1) - (24 h CONTROL-0 h CONTROL) denoted as “0 hrs–24 hrs”. Results were considered significant if p-value ≤ 0.05.

## Supplementary information

Supplementary_Figures_CD4_CD8

Supplementary Dataset 1

Supplementary Dataset 2

Supplementary Dataset 3

## Data Availability

The transcriptomic (RNA-Seq) data will be made available in the International Human Epigenome Consortium (IHEC) database in its next data release. Data will also be made available on request. The Illumina 450 K array data from CD4^+^ T cells, CD8^+^ T cells are available in the Gene Expression Omnibus (GEO) database under accession numbers GSE130029 and GSE130030 respectively.
