# Supplementary material for: Non-parametric combination analysis of multiple data types enables detection of novel regulatory mechanisms in T cells of multiple sclerosis patients
Source: Sci Rep. 2019 Aug 19;9:11996. doi: 10.1038/s41598-019-48493-7 (PMC6700160; doi:10.1038/s41598-019-48493-7)
Supplement: Supplementary file 1 — Supplementary_Figures_CD4_CD8 [file 41598_2019_48493_MOESM1_ESM.pdf]

Non-parametric combination analysis of multiple data types enables detection of novel regulatory mechanisms in T cells of multiple sclerosis patients

Sunjay Jude Fernandes[1,2,*], Hiromasa Morikawa[1,2,9], Ewoud Ewing[3], Sabrina Ruhrmann[3], Rubin Narayan Joshi[1,2], Vincenzo Lagani[4,5], Nestoras Karanthanasis[6,7], Mohsen Khademi[3], Nuria Planell Picola[11], Angelika Schmidt[1,2,8], Ioannis Tsamardinos[5,6], Tomas Olsson[3], Fredrik Piehl[3], Ingrid Kockum[3], Maja Jagodic[3], Jesper Tegnér[1,2,9] and David Gomez-Cabrero[1,2,10,11,*]

Affiliations:
1. Unit of Computational Medicine, Department of Medicine, Solna, Center for Molecular Medicine, Karolinska Institutet, Stockholm, Sweden.
2. Science for Life Laboratory, Solna, Stockholm, Sweden
3. Department of Clinical Neuroscience, Center for Molecular Medicine, Karolinska Institutet, Stockholm, Sweden
4. Institute of Chemical Biology, Ilia State University, Tbilisi, Georgia
5. Gnosis Data Analysis PC, Heraklion, Greece
6. Computer Science Department, University of Crete, Heraklion, Crete, Greece.
7. Computational Medicine Center, Thomas Jefferson University, 1020 Locust Street, Philadelphia, PA 19107 USA
8. Institute for Immunology, Biomedical Center, Ludwig-Maximilians-Universität, München, 82152 Planegg-Martinsried, Germany
9. Biological and Environmental Sciences and Engineering Division, Computer, Electrical and Mathematical Sciences and Engineering Division, King Abdullah University of Science and Technology, Thuwal, Kingdom of Saudi Arabia.
10. Mucosal and Salivary Biology Division, King's College London Dental Institute, London, SE1 9RT, United Kingdom.
11. Translational Bioinformatics Unit, Navarrabiomed, Complejo Hospitalario de Navarra (CHN), Universidad Pública de Navarra (UPNA), IdiSNA, Pamplona, Spain.
* Corresponding Authors

Correspondence should be addressed to,

Sunjay Jude Fernandes,
email: sunjay.fernandes@ki.se
Unit of Computational Medicine, L8:05,
Center for Molecular Medicine (CMM),
Karolinska University Hospital,
171 76 Stockholm, Sweden
Phone: +46727200505

AND

David Gomez-Cabrero
email: david.gomez.cabrero@navarra.es

Supplementary Figure 1

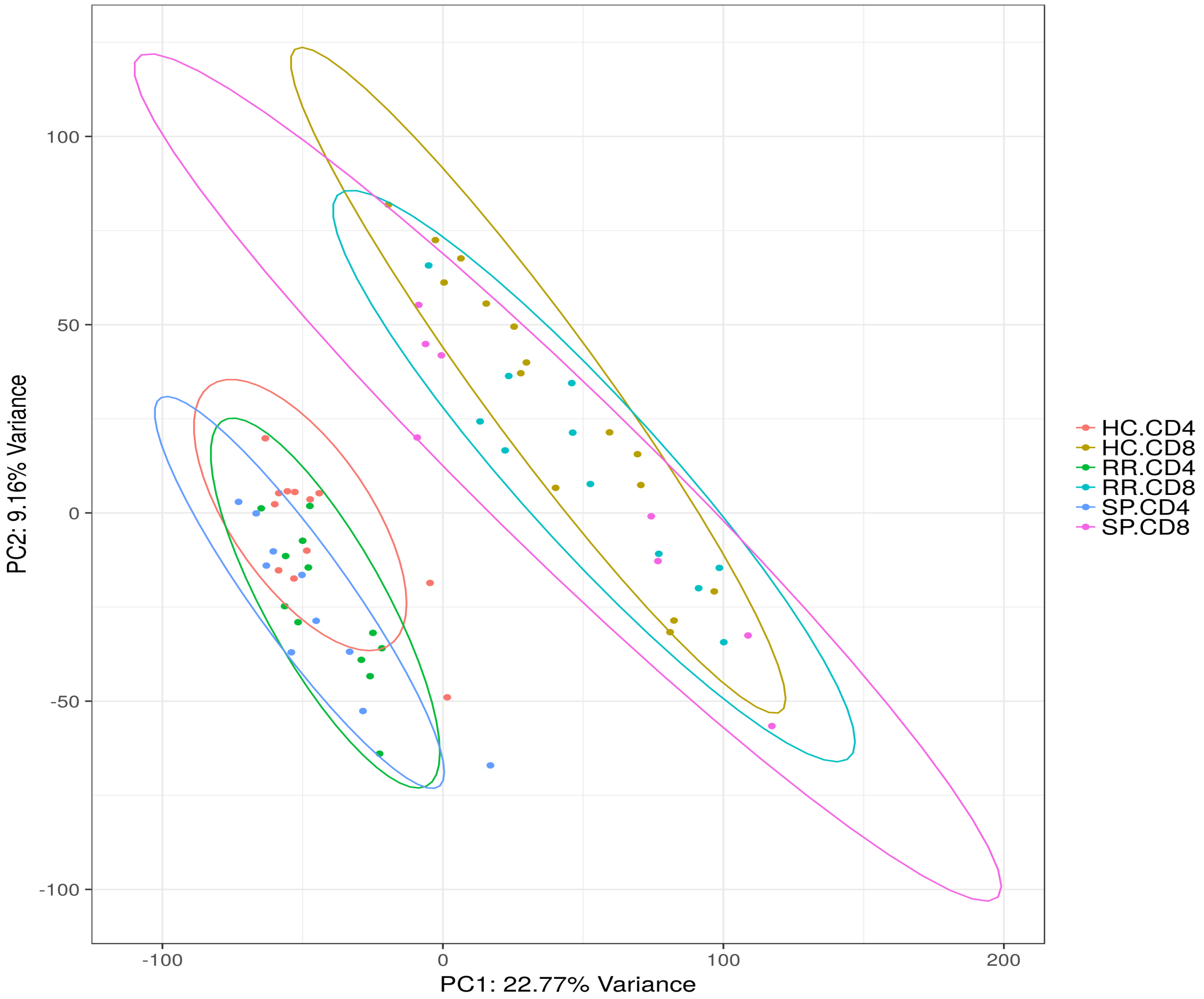

Principal component analysis (PCA) on genes detected in the transcriptomic data of CD4+ and CD8+ T cells for HC, RR and SP. A clear separation is seen between CD4 and CD8 while little separation is seen between HC and MS sample groups.

Supplementary Figure 2

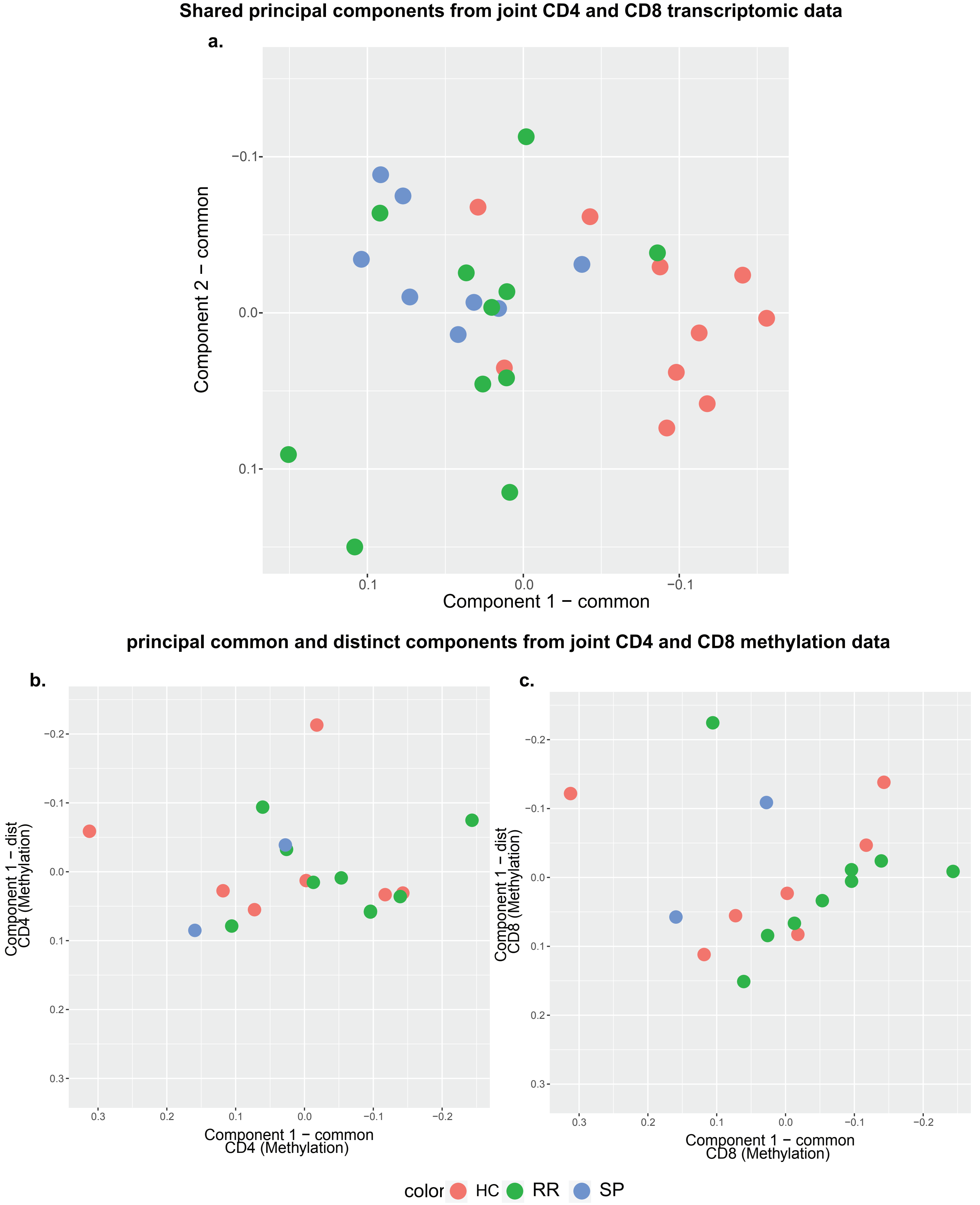

(**2a**) Gene expression, shared components. The figure depicts the shared first (x-axis) and second (y-axis) components of CD4 and CD8 gene expression data computed by JIVE. (**2b-c**) DNA Methylation, shared components. The panel **b** depicts the shared component of CD4 and CD8 DNA Methylation (x-axis) and CD4 distinctive component from DNA Methylation (y-axis) computed by JIVE. The panel **c** depicts the shared component of CD4 and CD8 DNA Methylation (x-axis) and CD8 distinctive component from DNA Methylation (y-axis) computed by JIVE. Healthy controls HC (red), RR (green), and SP (blue).

Supplementary Figure 3 (a-e)

Comparing ”Only paired" vs “All-samples" NPC strategies: To study if our NPC adaption to include non-paired samples added value, we ran NPC with "only paired" samples. Using the same dual cutoff of global p-value < 0.001 and FDR < 0.1, we obtained 30 genes. Of these 30 genes, 22 overlap with the analysis of "all-samples”. Hence, "all-samples” analysis identifies 127 additional significant findings when compared with the "only-paired" one. Next, we correlated (spearman) the unpermuted t-statistic obtained from the differential expression of "only-paired" vs “all-samples" for all genes found significant from both analysis (157 genes- 149 from the unpaired analysis and 30 from the paired analysis with 22 overlapping). This resulted in the following correlation: CD4: HCvsRR=0.977, RRvsSP=0.972 CD8: HCvsRR=0.978, RRvsSP=0.994 (a,b,c,d). Furthermore, we compared the p values and FDR of these 8 genes identified only from the "only-paired" analysis and we noted that these genes were very close to significance in the unpaired analysis (e). Finally, to ensure these 8 genes were not excluded from the unpaired analysis due to a bias introduced by unpaired samples, we plotted the expression of these individual genes per sample in the HC, RR and SP groups for CD4 and CD8 (f-u). We observed no bias was introduced from the unpaired samples. In conclusion, the addition of the unpaired samples provides additional power while not introducing any bias.

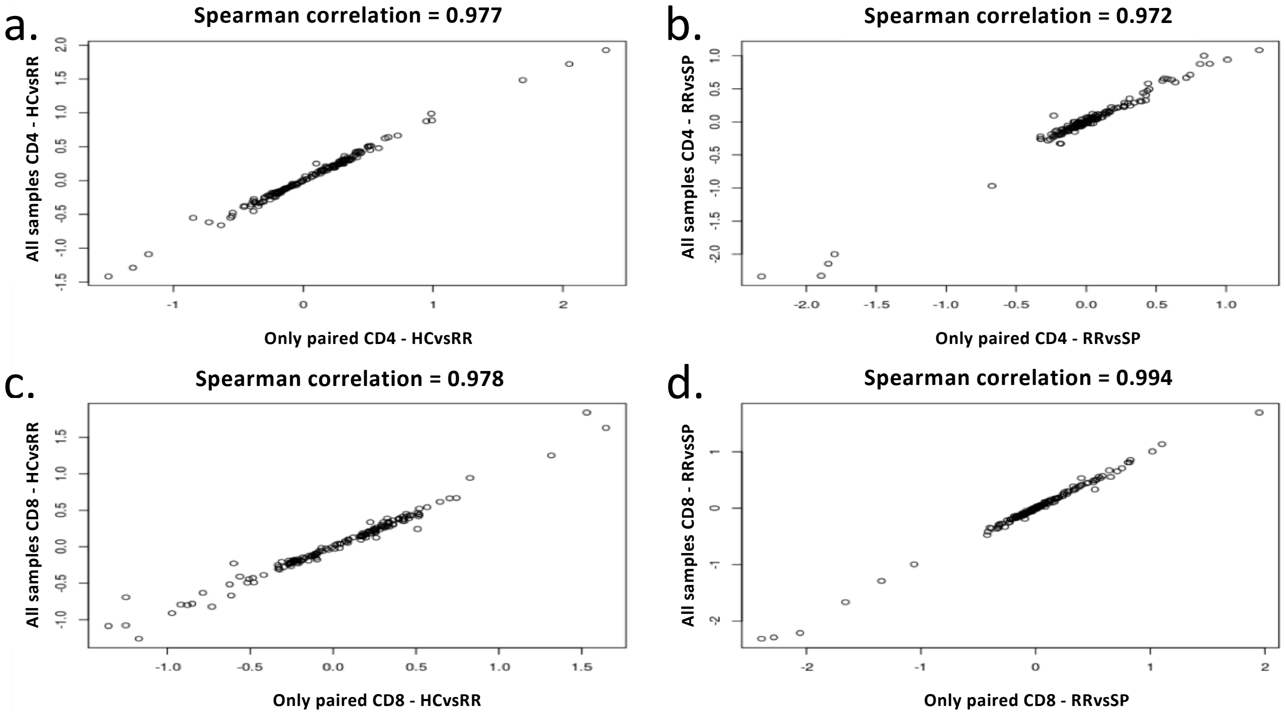

e.

Sl No	Gene name	Liptak - All samples	Liptak FDR - All samples	Liptak - Only paired	Liptak FDR – Only paired
1	UBE2D1	0,000150	0,090040	0,000850	0,149667
2	SSBP1	0,000150	0,053070	0,000650	0,100615
3	TXNIP	0,000350	0,068799	0,001650	0,154911
4	SSH2	0,000150	0,059573	0,001050	0,069740
5	AASDH	0,000050	0,058171	0,000450	0,100234
6	TYW5	0,000350	0,097376	0,001350	0,150135
7	ZNF583	0,000050	0,058709	0,000350	0,116606
8	MARS2	0,000050	0,051029	0,001950	0,169884

Supplementary Figure 3 (f-m)

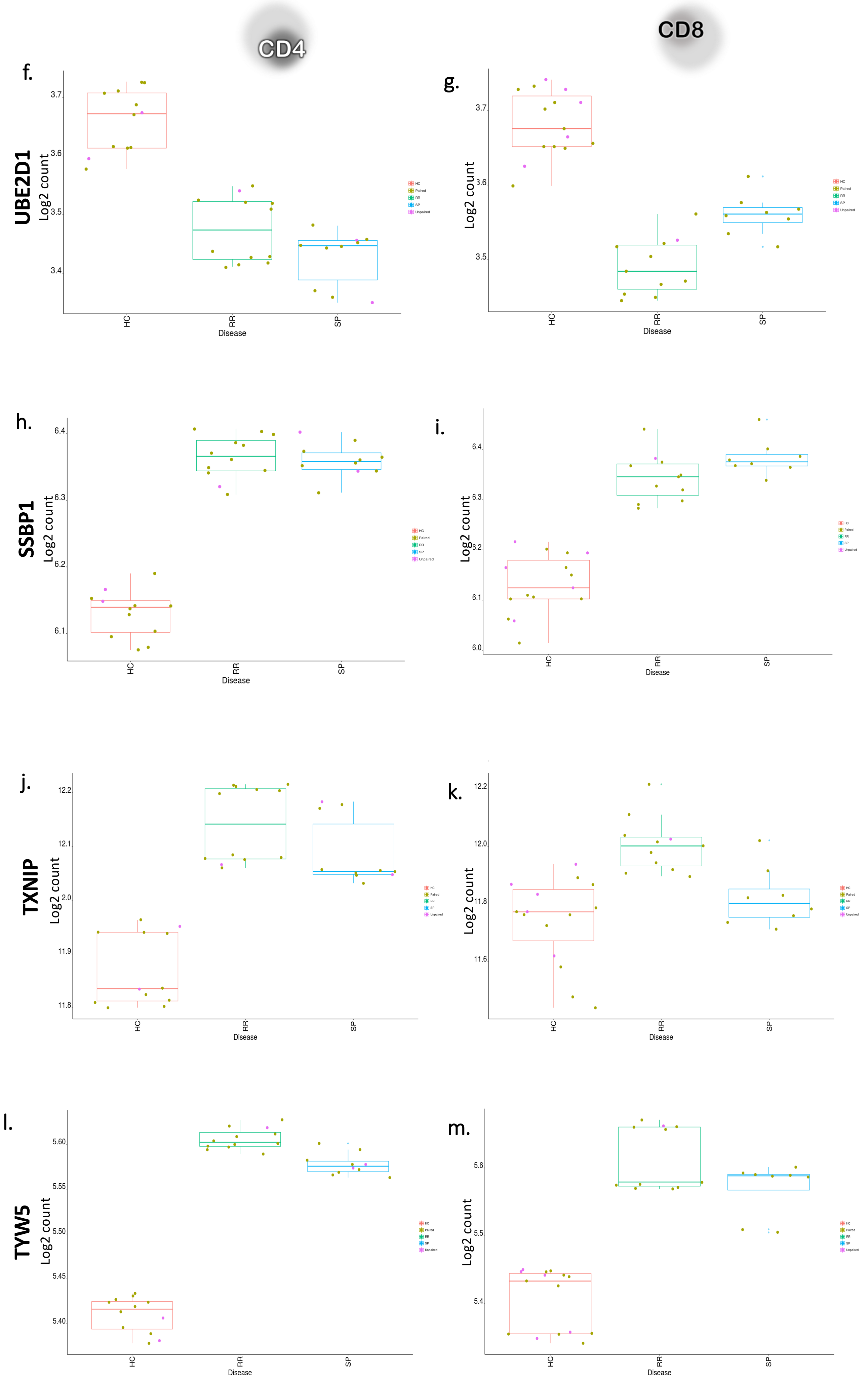

Supplementary Figure 3 (n-u)

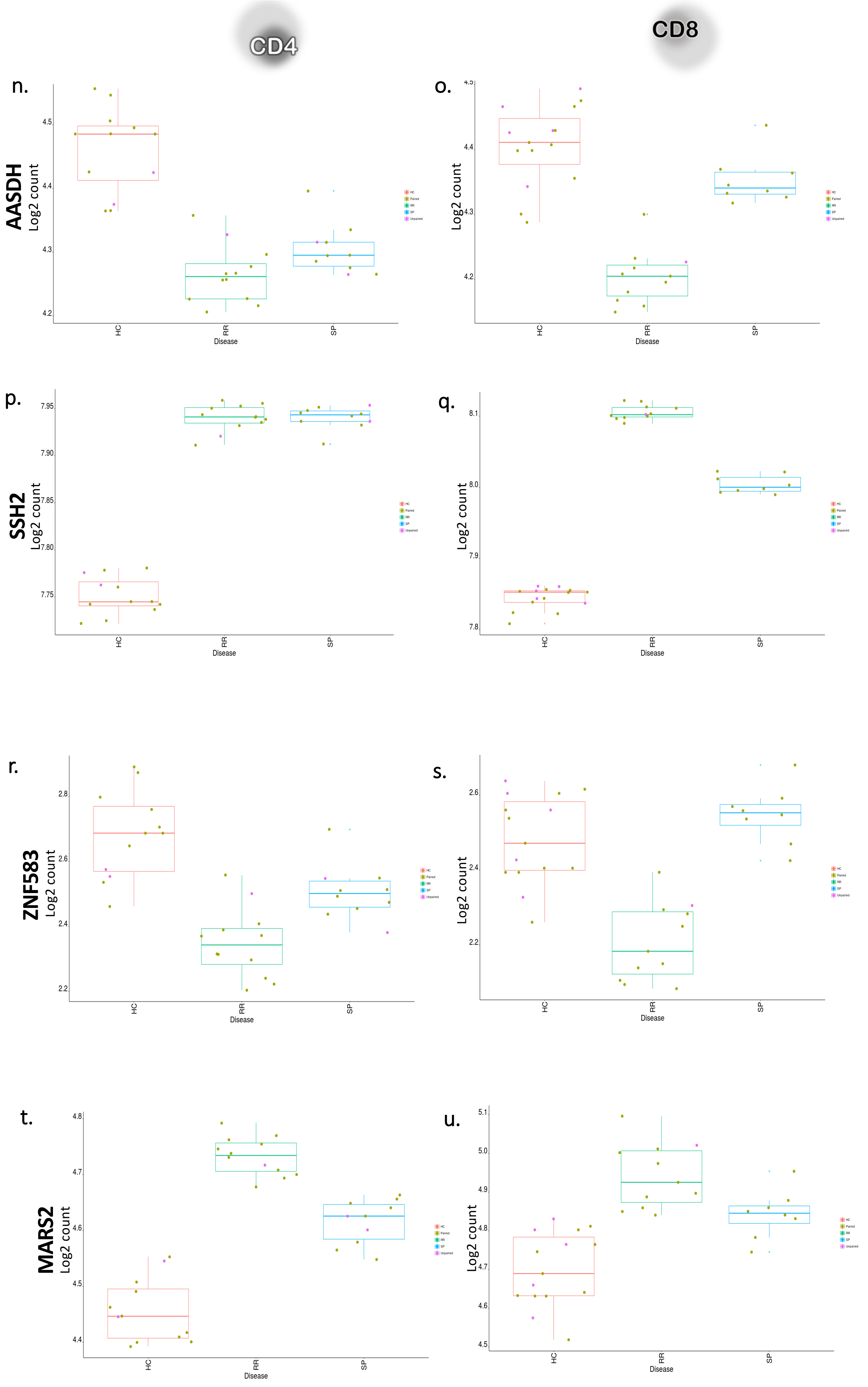

Supplementary Figure 4

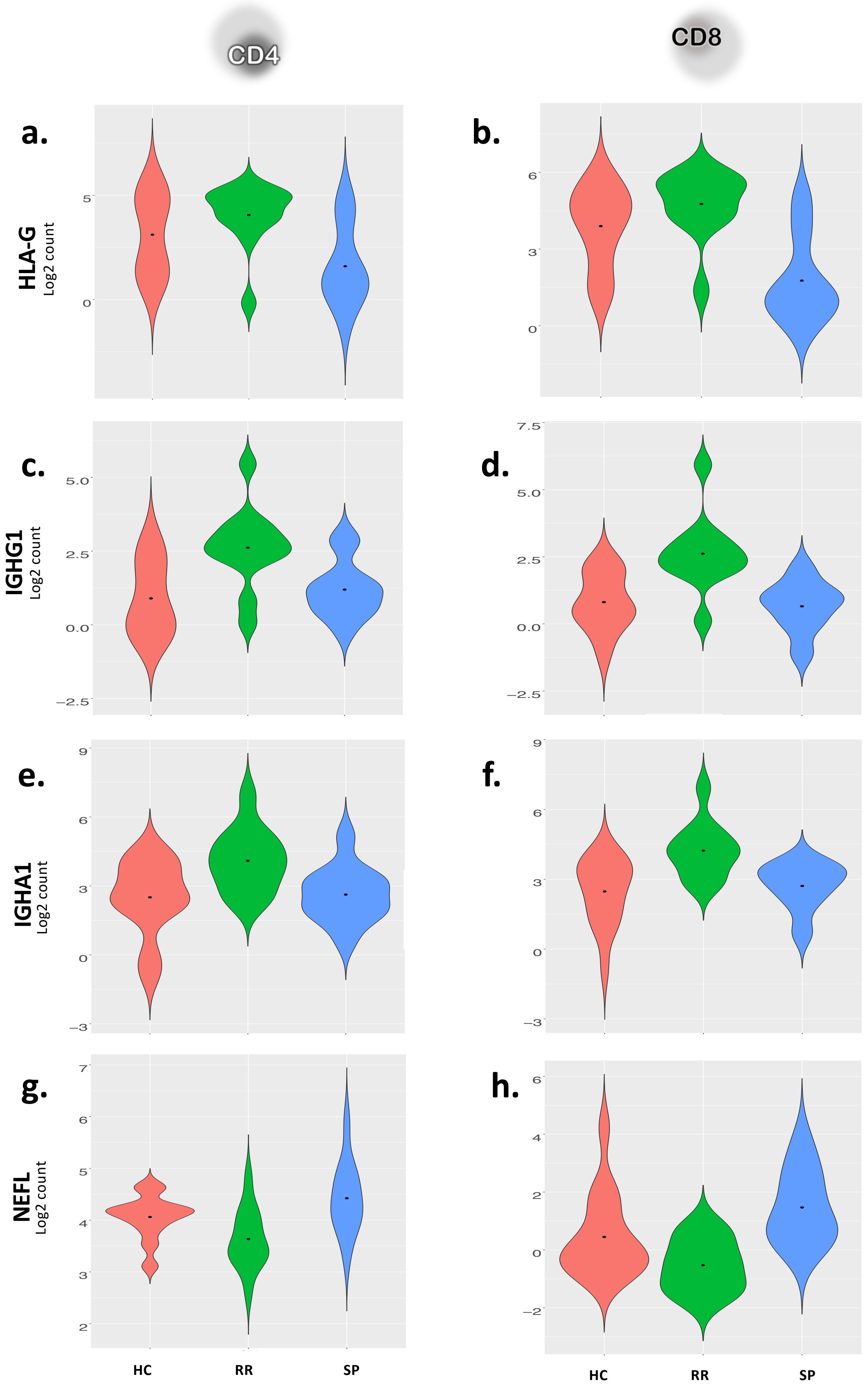

Normalized log2 count of genes expressed in CD4+ and CD8+ T cells in HC, RR and SP showing changes in the trend of expression.
